# First person – Ayyappa Raja Desingu Rajan

**DOI:** 10.1242/dmm.052167

**Published:** 2024-12-04

## Abstract

First Person is a series of interviews with the first authors of a selection of papers published in Disease Models & Mechanisms, helping researchers promote themselves alongside their papers. Ayyappa Raja Desingu Rajan is first author on ‘ Generation of a zebrafish neurofibromatosis model via inducible knockout of *nf2a/b*’, published in DMM. Ayyappa Raja is a postdoctoral researcher in the lab of Marianne Bronner at California Institute of Technology, Pasadena, CA, USA. Using the neural crest as a model, Ayyappa Raja investigates lineage decisions that contribute to specific tissue types, such as the meninges, and how developmental gene programs can go array in cancers such as meningiomas and neurofibromatosis.


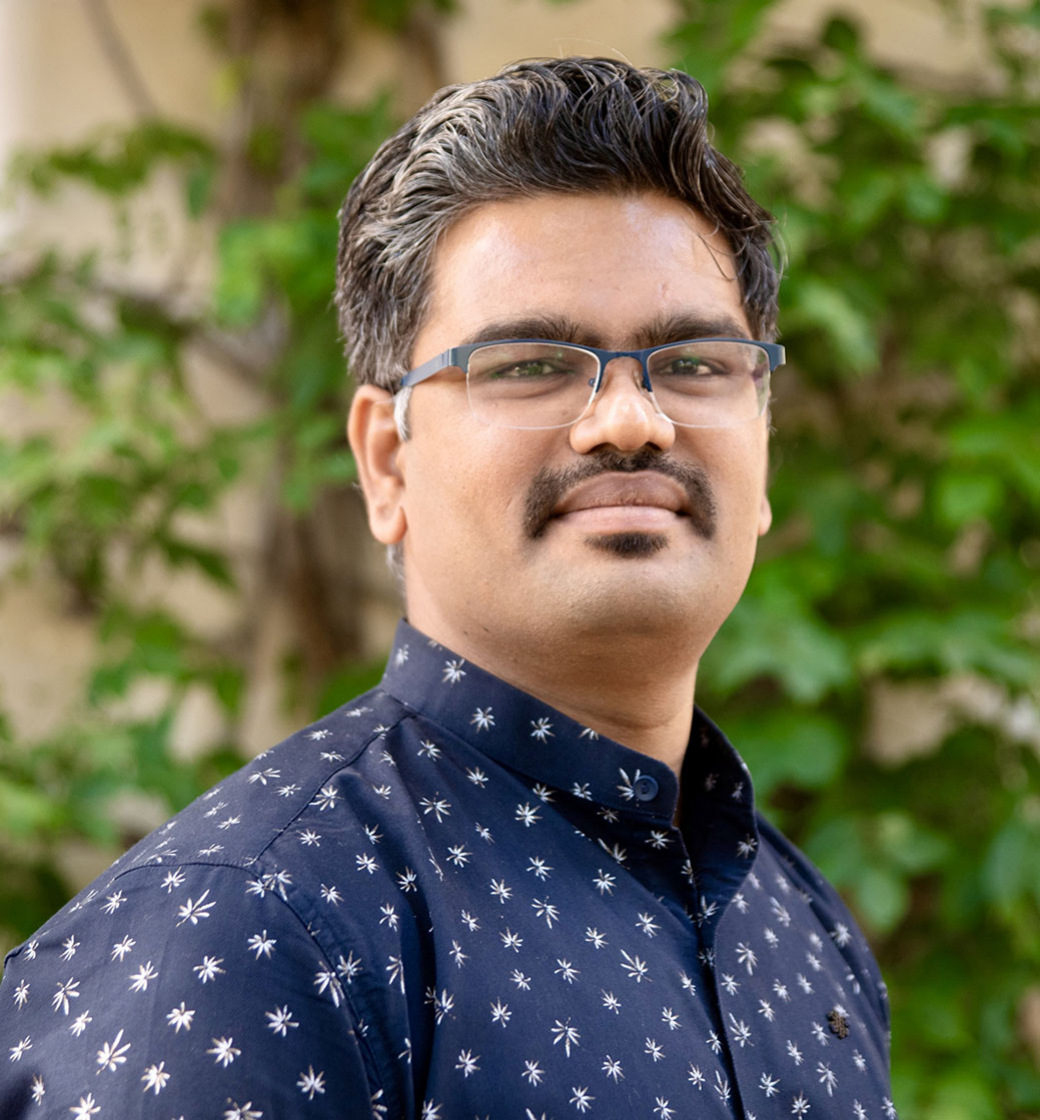


**Ayyappa Raja Desingu Rajan**

**Who or what inspired you to become a scientist?**

I vividly remember my first experience of being fascinated by biology when I was in grade 3 and had a peek at formalin-fixed animal embryos in glass jars. What are they? How do they form? These questions led me to study zoology as an undergraduate, focusing on animal development. As a first-generation college student, I was captivated by developmental biology, genetics and molecular biology courses. I discovered my passion for research as an intern in a major laboratory, which inspired me to be a scientist.

[…] we generated an inducible zebrafish model that mirrors the tumour manifestations observed in patients with NF-2.

**What is the main question or challenge in disease biology you are addressing in this paper? How did you go about investigating your question or challenge?**

Neurofibromatosis type 2 (NF-2) is an autosomal-dominant disorder resulting from germline/mosaic mutations in the *NF2* tumour suppressor gene, leading to multiple benign tumours in the nervous system and along peripheral nerves. Despite its benign nature, NF-2-associated tumours can lead to neurological deficits such as early-onset hearing loss, issues with balance, cataracts, seizures, pain and problems with facial expressions. In this study, we generated an inducible zebrafish model that mirrors the tumour manifestations observed in patients with NF-2.

**How would you explain the main findings of your paper to non-scientific family and friends?**

Modelling the multitude of phenotypes seen in NF-2 patients has been challenging, as deletion of both copies of *Nf2* in mice is lethal and demonstrates a tumour spectrum that differs significantly from that observed in NF-2 patients. To circumvent this, we generated a novel zebrafish model that mimics the complexities of the human NF-2 disorder. We show that *nf2a/b* is expressed in the neural crest, meninges and Schwann cells during early development and in adult Schwann cell precursors. Loss of *nf2a/b* results in aberrant proliferation of these cell types, eventually leading to Schwannomas, meningiomas, cataracts and abnormal pigmentation. Since our inducible model can undergo conditional inactivation of *nf2a/b* over various ages, this model can potentially recapitulate neurofibromatosis onset at different stages of life.

**What are the potential implications of these results for disease biology and the possible impact on patients?**

Our model promises to enable the testing of therapeutic agents or large-scale chemical libraries for the ability to ameliorate the phenotypes of NF-2. The results demonstrate the utility of this model for recapitulating a broad range of phenotypes associated with NF-2 that closely mimic human disease. The accessibility, ease of manipulation, availability of genetics and facility of imaging promise to make this a handy model for further exploration of tumour ontogeny and assaying means of treatment.

**Figure:**
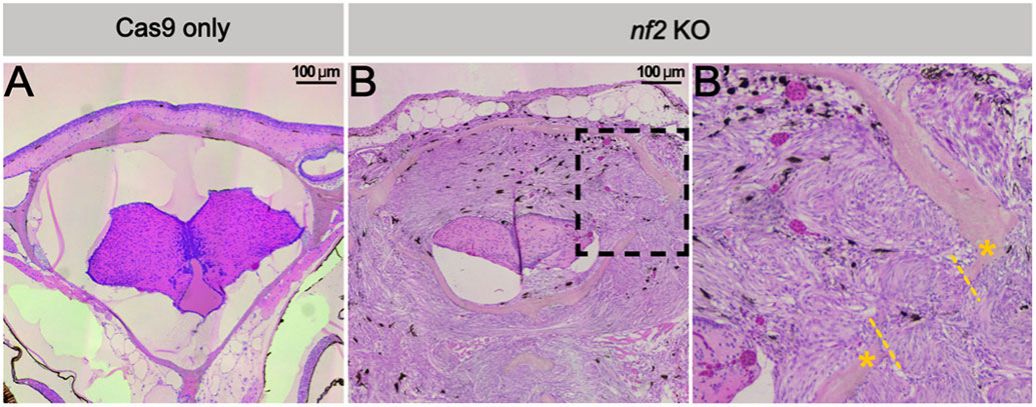
***nf2a/b* knockout adult zebrafish develop meningiomas.** (A-B′) Transverse sections of *nf2a/b* knockout adult zebrafish displaying meningioma (B,B’) compared to Cas9-only control (A). The black dashed line box indicates the zoomed region. The region between the yellow dashed lines shows meningioma penetrating the cranium. Yellow asterisks indicate cranium.

**Why did you choose DMM for your paper?**

My first publication was in The Company of Biologists’ journal Development. I loved the review and publication handling process. When we completed our manuscript, my mentor and I discussed where to send it and decided on DMM, which we thought would be a great fit. Being open access and the Read & Publish initiative tipped the scales heavily in favour of DMM. In my opinion, everyone should consider publishing in DMM. It's great.

[…] science is a global phenomenon, and a lack of funding opportunities should not hinder its progress.

**Given your current role, what challenges do you face and what changes could improve the professional lives of other scientists in this role?**

As a first-generation graduate, I moved to the USA for my postdoctoral studies. While the scientific atmosphere is excellent here, I feel that funding opportunities are limited for non-US citizens. Funding agencies should look into generating opportunities for new immigrant postdocs, especially in the basic sciences. Having their own funding will boost the morale of postdocs and provide stability to their career. After all, science is a global phenomenon, and a lack of funding opportunities should not hinder its progress.

**What's next for you?**

Following my postdoctoral research, I want to establish my independent research laboratory. When I envision my future lab, I see it at the interface of developmental biology and cutting-edge molecular biology techniques to answer wide-ranging questions in stem cell biology and tumorigenesis. My mentor, Professor Marianne Bronner, has taught me the significant impact of a positive work environment on the wellbeing and productivity of team members. Learning from this, I aim to establish a research group dedicated to delivering high-quality outcomes while nurturing an inclusive and collaborative atmosphere for graduate and undergraduate students from diverse backgrounds.

**Tell us something interesting about yourself that wouldn't be on your CV**

I love hiking with my family and playing cricket, and I am a huge fan of the manga/anime ‘One Piece’. As a scientist, your brain is tuned to think about grants/projects/experiments 24×7. These activities let me focus on the present and just enjoy the moment.
